# The influence of arbuscular mycorrhizal fungi inoculation on yam (*Dioscorea* spp.) tuber weights and secondary metabolite content

**DOI:** 10.7717/peerj.1266

**Published:** 2015-09-24

**Authors:** Fun-Chi Lu, Chen-Yu Lee, Chun-Li Wang

**Affiliations:** Department of Plant Industry, National Pingtung University of Science and Technology, Pingtung, Taiwan

**Keywords:** Yams (*Dioscorea* spp.), Arbuscular mycorrhizal fungi (AMF), Secondary metabolites, Microbial fertilizers

## Abstract

Arbuscular mycorrhizal fungi (AMF) are widely distributed in nature. They live in the roots of higher plants, in a symbiotic relationship. In this study, five commercial species of yams (*Dioscorea* spp.) were inoculated with six species of AMF, *Glomus clarum*, *G. etunicatum*, *G. fasciculatum*, *Gigaspora* sp., *G. mosseae*, and *Acaulospora* sp., in field cultivation conditions to investigate the influence of AMF inoculation on tuber weights and secondary metabolite content in yam tubers. The results showed that mycorrhizae formation rates ranged from 63.33% to 90%. *G. etunicatum* inoculation treatment increased the tube weights of the five species of yam tubers by 39%, 35%, 20%, 56%, and 40% for Tainung 1, Tainung 2, Ercih, Zihyuxieshu, and Tainung 5, respectively. The content of secondary metabolites, such as polyphenols, flavonoids, and anthocyanin, was significantly increased by the AMF treatment in tuber flesh and peel of all the tested yam species. Specifically, the maximums exchange of secondary metabolite contents increased to 40%, 42%, and 106% for polyphenols, flavonoids, and anthocyanin, respectively, in the tuber fresh. This study revealed that different species of yam had varying degrees of affinity with various AMF species; selecting effective AMF species is necessary to facilitate yam growth and improve the quality and quantity of yam tubers.

## Introduction

The symbiotic association between arbuscular mycorrhizal fungi (AMF) and the roots of plants is widespread in the natural environment. AMF establish symbiosis with approximately 80% of the vascular plant species in all terrestrial biomes (Smith et al., 2010). AMF are of great ecological importance, because arbuscular mycorrhizae are involved in the most widespread form of plant symbiosis and often improve plant productivity (Fedderman et al., 2010). AMF benefit their host principally by increasing the uptake of relatively immobile phosphate ions (Miransari et al., 2009). Other benefits include: increased uptake of macronutrients, including N, K, and Mg (Veresoglou, Shaw & Sen, 2010), as well as some micronutrients (Kim et al., 2009); increased resistance to soil pathogens (Lingua et al., 2002); increased tolerance to salinity, heavy metals, and drought stress (Li et al., 2011); regulation of the synthesis and distribution of plant hormones (Barker & Tagu, 2000); and maintenance of soil aggregate stability (Degens, Sparling & Abbott, 1996).

The yam (*Dioscorea* spp.) is a perennial vine plant with tubers. Because yam tubers are edible, medicinal, and used as health foods, they have received considerable attention among the medicinal plants (Olubobokun et al., 2013). In recent years, people have increasingly valued natural medicinal plants as well as medicinal resources. Yams are rich in polyphenols (Aarti et al., 2013), which play an essential role in antioxidant and biological activity (Kalt et al., 1999). Studies have indicated that polyphenolic compounds are positively correlated with antioxidant capacity (Yang et al., 2012) and that the antioxidant activity of flavonoids can eliminate reactive oxygen species and terminate the chain reactions of free radicals (Arora, Nair & Strasburg, 1998). Moreover, purple yams contain abundant anthocyanins, which have the antioxidant capacity to inhibit lipid peroxidation, resist the attack of free radicals, diminish low-density lipoproteins (LDLs), and reduce the occurrence of cardiovascular diseases (Wang, Cao & Prior, 1997); thus, the antioxidant substances that exist in yam tubers have potential for development and utilization in medicine (Teixeira et al., 2013).

AMF establish a symbiotic relationship with plant roots and influence the host’s metabolic processes. Numerous studies have noted that AMF can directly or indirectly influence the secondary metabolism of plants, causing changes in secondary metabolite levels (Araim et al., 2009; Yaghoub & Weria, 2013). For example, AMF can induce the synthesis of caffeic acid and rosmarinic acid in the medicinal plant sweet basil (*Ocimum basilicum*) (Toussaint, Smith & Smith, 2007). In addition, the inoculation of *Glycyrrhiza uralensis* Fisch with AMF can prompt the accumulation of glycyrrhizin containing triterpenoid saponin (Liu et al., 2007). AMF inoculation also stimulates the production of camptothecin in the seedlings of *Camptotheca acuminata* (Zhao, Wang & Yan, 2007).

AMF inoculation not only promotes the growth of medicinal plants but also improves the productivity and quantity of chemicals (Karthikeyan, Joe1 & Jaleel, 2009). The edible part of the yam is the tuber, which produces substantial amounts of secondary metabolites that have medicinal value. However, no studies have investigated the inoculation of yams with AMF. Interaction of yams and AMF may be able to improve the tuber yield and food value. Thus, in this study we inoculated yams with six species of AMF. The tuber weights and the polyphenols, flavonoids, and anthocyanins in these yams were analyzed. The results can be used as a basis for selecting symbiotic AMF species suitable to enhance the application and value of medicinal yams.

## Materials and Methods

Five species of yams were used in the experiment: three white flesh yams (Tainung 1, Tainung 2, and Ercih) and two purple flesh yams (Zihyuxieshu and Tainung 5) provided by yam farmers in Nantou, Taiwan. Six species of AMF were used: *Glomus clarum* (Gc), *G. etunicatum* (Ge), *G. fasciculatum* (Gf), *G. mosseae* (Gm), *Gigaspora* sp. (Gg), and *Acaulospora* sp. (Asp), isolated and identified by the laboratory of plant physiology and value-added microorganism, National Pingtung University of Science and Technology (NPUST) all of which were cultured with bahiagrass root for 6 months. The roots and rhizosphere substrate were harvested and used as inoculums.

### Field experiment

In April 2013, the five species of yam seedlings were induced to sprout. After 30 days, the five yam species sprouts that had grown to 30 cm were inoculated with one of six AMF species separately. Each sprout received 2 mL of inoculant containing about 300 spores. Noninoculated yams of each of the five species were used as the control groups. The inoculated yams were planted 250 × 30 cm apart from one another on loam soil with a pH of 7.2 and electrical conductivity (EC) of 1.2 mS/cm in the farm of NPUST. A split-plot design was conducted in seven treatments, and five repetitions were tested for each treatment. The annual average temperature and amount of sunlight were about 24.5 °C and 11.93 MJ/m^2^, respectively.

### Investigation of mycorrhizal formation rate

Six weeks after AMF inoculation, the roots of inoculated yams were collected. The structure of symbiotic mycorrhizae was observed using an optical microscope. Three repetitions for each treatment and 10 plant root sections (1–1.5 cm) for each repetition were examined. The mycorrhizal formation rates of the various AMF species were calculated by using the percentage of the root segments that were colonized by AMF (Koske & Gemma, 1989).

### Harvest of yam tubers

Tubers of the five yam species, each inoculated with six AMF species, were harvested during the late stage of the growth period in December 2013. The harvested tubers were weighed to compare the yield among the various treatment groups.

### Determination of the secondary metabolites in yam tubers

#### Yam tuber preparation

The peel and sliced flesh of the harvested yam tubers were dried in an oven at 40 °C for 72 h, after which they were ground (Yuqi, DM-6, Taiwan), passed through a 60 mesh sieve and stored at 4 °C until further use. We then mixed 5 g of yam powder with 100 mL of methanol solution for reflux extraction (BC-2D; Taiwan Hipoint Corporation, Kaohsiung City, Taiwan) at 85 °C for 2 h. The extract solution was vacuum filtered, and 100 mL of the methanol solution was added to the filtered residue for one more extraction. The filtered extract solution was filtered again through filter paper (Whatman No.1), concentrated, and dried using a rotary evaporator (Laborota 4000; Heidolph Instruments, Schwabach, Germany). The resulting crystalline solids were weighed and dissolved in the methanol solution for subsequent usage.

#### Determination of total polyphenol content

The methanol extract from the yam tuber peel and flesh (0.1 mL) was taken to interact with 1 mL of Folin–Ciocalteu reagent for 5 min, after which 0.9 mL of 20% sodium carbonate was added. After a standing time of 10 min, the solution was centrifuged at 3,000 rpm for 10 min, and the absorbance of the supernatant was measured at 735 nm by spectrophotometer (Hitachi, U-2800). The standard curve of gallic acid solution was used to determine the total polyphenol content (Sato et al., 1996).

#### Determination of flavonoid content

Yam tuber peel and flesh methanol extract (0.2 mL) was added to 1.5 mL of deionized water, followed by 0.1 mL of 10% Al(NO_3_)^3^ and 0.1 mL of 1 M CH_3_COOK. The mixture was placed in the dark for 40 min, and the absorbance was measured at 415 nm (Jia, Ma & Wang, 1990). The standard curve of quercetin was adopted to determine the flavonoid content.

#### Determination of anthocyanin content

Yam tuber peel and flesh powder (0.5 g) was added to 10 mL of l% HCl/MeOH and thoroughly mixed; the mixture was placed in the dark at 4 °C for 24 h, after which it was centrifuged at 4 °C, 1,000 × *g*, for 15 min (Padmavati et al., 1997). The absorbance of the supernatant was measured at 530 nm and 657 nm. The anthocyanin content (µmole/g) = (A530–0.33 × A657)÷31.6 × milliliter ÷ gram.

### Statistic analysis

Data were analyzed by one way ANOVA using SAS 9.1 with variance analysis conducted for all test data. THE difference between treatments was determined using and Duncan’s Multiple Range Test (*P* < 0.05).

## Results and Discussion

### Mycorrhizal formation rate

The mycorrhizal formation rate was measured for five species of yams inoculated with six species of AMF (Table 1). The results demonstrated that AMF had infected the plant roots, with mycorrhizae formation rates from 63.3 to 90.0%. The hyphae extended between cortex cells and produced vesicles, and the extracellular hyphae formed spores (data not shown). All six AMF species were able to form a symbiotic relationship with the host yams, indicating that these AMF were high compatible with the host yams.

**Table 1:** The mycorrhizal formation rate (%) of *Dioscorea* spp. after inoculation with six species of AMF for six weeks. The mycorrhizal formation rates of the various AMF species were calculated by using the percentage of the root segments that were colonized by AMF. *n* = 30.

Mycorrhizal formation rate (%)					
AMF species	Tainung 1	Tainung 2	Ercih	Zihyuxieshu	Tainung 5
Gc	66.7 ± 2.3	73.3 ± 2.1	86.7 ± 1.5	66.7 ± 2.1	73.7 ± 1.5
Ge	86.7 ± 1.5	83.3 ± 1.2	86.7 ± 1.2	88.3 ± 0.6	80.7 ± 2.0
Gf	73.3 ± 1.5	90.0 ± 1.7	80.0 ± 2.0	86.7 ± 1.5	76.7 ± 2.3
Gg	76.7 ± 2.5	83.3 ± 0.6	73.3 ± 1.5	80.3 ± 1.7	86.7 ± 1.5
Gm	90.0 ± 1.7	86.7 ± 2.3	66.7 ± 2.1	86.7 ± 1.5	63.3 ± 1.5
Asp	86.7 ± 1.5	70.0 ± 2.0	86.7 ± 1.5	86.7 ± 0.6	73.3 ± 1.2
Control	6.7 ± 0.6	6.7 ± 0.6	13.3 ± 0.6	10.0 ± 1.0	13.3 ± 0.6

**Notes.**

AMF species Gc
*Glomus clarum*
Ge
*G. etunicatum*
Gf
*G. fasciculatum*
Gg *Gigaspora* sp. Gm
*G. mosseae*
Asp *Acaulospora* sp

### Tuber yield in the field experiment

**Figure 1:**
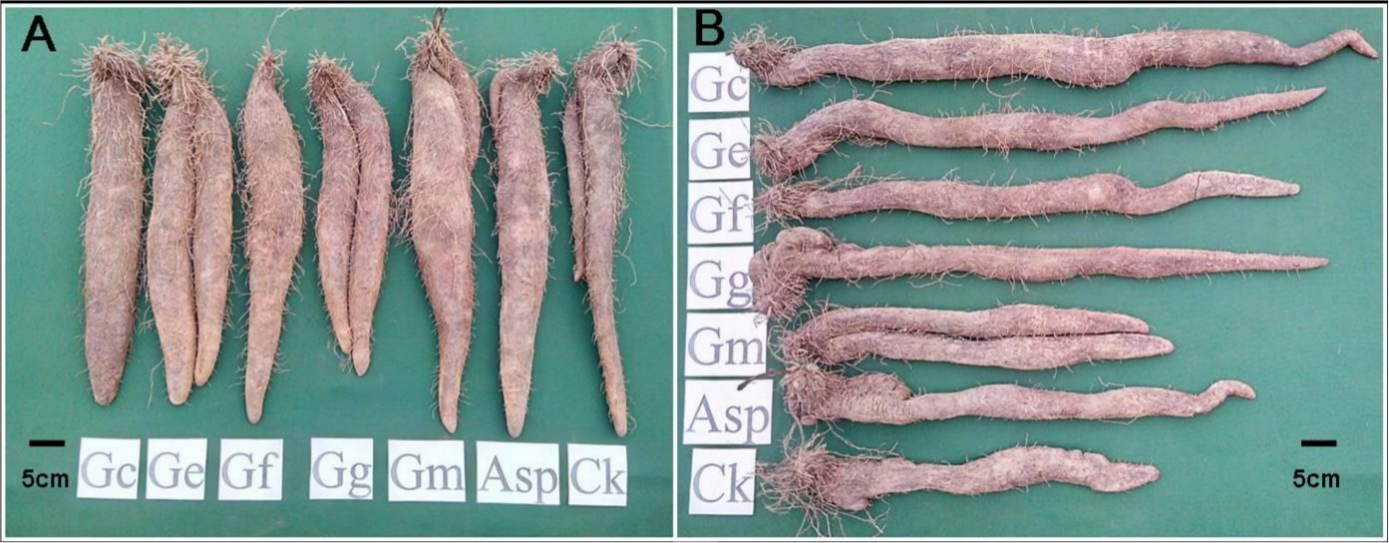
The tuber growth and development of *Dioscorea* spp. inoculated with six AMF species for eight months. (A) Ercih line. (B) Tainung 5. AMF species Gc, *Glomus clarum*; Ge, *G. etunicatum*; Gf, *G.fasciculatum*; Gg, *Gigaspora* sp.; Gm, *G.mosseae* and Asp, *Acaulospora* sp.

The comparison of tuber weights and size between the AMF-inoculated and noninoculated yams after 8 mo cultivation demonstrated improved tuber when inoculated with AMF (Fig. 1 and Table 2). The tuber weights of Tainung 1 and Tainung 2 in the treatment groups were generally greater than the weights of the control groups. The weights of Tainung 1 and Tainung 2 inoculated with Asp (1,490 g/plant and 1,610 g/plant) were approximately equal to those of the control groups (1,460 g/plant and 1,640 g/plant). The tuber weights of Tainung 1 inoculated with Gc, Ge, and Gm and those of Tainung 2 inoculated with Gc, Ge, Gf, Gg, and Gm were significantly greater than the weights of the control group (*P* < 0.05). Moreover, except for the yield of Ercih treated with Gg (1,430 g/plant), which was lower than that of the control group (1,570 g/plant), the weights of the other treatment groups were greater than those of the control groups. The increases in the weights of the Ge (1,890 g/plant) and Gm (1,840 g/plant) treatment groups were the most statistically significant (*P* < 0.05).

**Table 2:** Five commercial species of yams (*Dioscorea* spp.) were inoculated with six species of AMF. Comparison of tuber weight of *Dioscorea* spp. after 8 mo inoculation with AMF species, as indicated, compared to control (not inoculated).

Tuber weight (g/plant)										
AMF species	Tainung 1		Tainung 2		Ercih		Zihyuxieshu		Tainung 5	
	Tuber weight	Increased rate (%)	Tuber weight	Increased rate (%)	Tuber weight	Increased rate (%)	Tuber weight	Increased rate (%)	Tuber weight	Increased rate (%)
Gc	1,930ab^*^	32.3	2,120b	29.5	1,780ab	13.3	1,840ab	59.4	1,220a	59.7
Ge	2,040a	39.5	2,210ab	35.0	1,890a	20.4	1,810ab	56.5	1,070ab	40.1
Gf	1,710bcd	17.3	2,480a	51.5	1,690ab	8.0	1,540bc	33.3	870b	13.9
Gg	1,540cd	5.3	2,160ab	31.9	1,430b	−8.5	2,000a	73.5	1,040ab	36.4
Gm	1,750bc	20.1	2,370ab	44.8	1,840a	17.3	1,220cd	5.9	980ab	28.3
Asp	1,490d	1.9	1,610c	−1.8	1,620ab	3.2	1,140d	−1.4	820b	7.1
Control	1,460d		1,640c		1,570ab		1,150d		760b	

**Notes.**

AMF species Gc
*Glomus clarum*
Ge
*G. etunicatum*
Gf
*G. fasciculatum*
Gg *Gigaspora* sp. Gm
*G. mosseae*
Asp *Acaulospora* sp

* Columns with a different letter on are significantly different at *P* < 0.05, *n* = 5.

For the purple yams, the tuber weights of Asp-treated Zihyuxieshu was lower than that of the control group (1,140 g/plant and 1,150 g/plant, respectively), whereas the weights of the other treatment groups were all higher (Table 2). The tuber weights of Zihyuxieshu treated with Gc, Ge, Gf, and Gg were significantly greater than the weights of the control group (*P* < 0.05). In addition, the tuber weights of Tainung 5 in all the treatment groups were greater than that of the control group, but only the weights of the Gc-treated group (1,220 g/plant) were significant (*P* < 0.05).

Studies have demonstrated that AMF positively influences plant growth in crops that are harvested for underground parts, such as cassava, potato, and sweet potato (Don-Rodrigue et al., 2013). In tuber crops, many studies have demonstrated the enhancement of growth and yield by AMF. Recent studies highlighted potential of AMF for yam productivity improvement. AMF colonization was reported to increase yam yields in Nigeria (Oyetunji & Afolayan, 2007) and in the ‘Yam belt’ of Benin (Tchabi et al., 2010). AMF diversity was recently estimated in soils from yam cropping fields in Nigeria (Dare et al., 2013).

Our study showed that AMF inoculation had enhanced or diminished effects on the tuber weights of five commercial yams currently cultivated in Taiwan. AMF Gc, Ge, and Gm induced a significant weight increase in white yam tubers. Gc, Ge, and Gg had similar effects on purple yam tubers. However, inoculation with AMF Gg and Asp had negative impact in tuber weight. A though the AMF association can offer multiple benefits to the host plant it may not be obviously mutualistic at all points in time, and it is possible under some conditions that the AMF may subvert their host plant into supplying carbon with no apparent benefit to the plant. In some cases, this can cause a decline in growth (Lerat et al., 2003). It may be that plants and fungi compete for nutrients when they are limiting, and some fungi are able to sequester nutrients in fungal tissue. Another explanation is that changes within the plant due to AM fungal colonization may render plants less able to take up certain nutrients via systemic changes in plant chemistry and/or gene expression (Hodge, Helgason & Fitter, 2010).

There is mounting evidence for functional specialization among AMF (Powell et al., 2009; Thonar et al., 2011). This specialization can result in functional complementarity among AMF in communities, where a diverse community of fungi may confer additional benefits to host plants (Sikes, Powell & Rillig, 2010). This specialization may result from the various degrees of symbiotic compatibility between AMF species and the hosts. Thus, screening to determine the optimal AMF species for hosts is essential for promoting the growth of yams and tuber weights.

### Total polyphenol content

The tuber polyphenol content in the methanol extracts from three species of white flesh yams inoculated with six species of AMF was measured (Table 3). In the white yams, the polyphenol content in the methanol extracts from the tuber flesh in several treatment groups was statistically significant (*P* < 0.05), particularly for Tainung 1 treated with Ge (44.5 mg/g) and Asp (43.3 mg/g), Tainung 2 treated with Ge (34.0 mg/g) and Gf (38.3 mg/g), and Ercih treated with Gf (16.9 mg/g) and Gm (15.8 mg/g). Moreover, the polyphenol content in the tuber peel of the three white flesh yams was higher than that in the tuber flesh. As the polyphenol content in the tuber flesh increased, the content in the tuber peel also increased (Table 3).

**Table 3:** Three commercial species of white flesh yams (*Dioscorea* spp.) were inoculated with six species of AMF. Polyphenol content of methanolic extracts from three species of white flesh yams (*Dioscorea* spp.) flour inoculated with six species of AMF.

Polyphenol content of yams methanolic extract (mg/g)															
AMF species	Tainung 1					Tainung 2					Ercih				
	Tuber flesh	Increased rate (%)	Tuber peels	Increased rate (%)	Peels/Flesh ratio	Tuber flesh	Increased rate (%)	Tuber peels	Increased rate (%)	Peels/Flesh ratio	Tuber flesh	Increased rate (%)	Tuber peels	Increased rate (%)	Peels/Flesh ratio
Gc	30.3b^*^	−2.0	49.8b	13.7	1.6	19.8e	−33.3	43.9e	−6.9	2.2	11.1d	−22.2	40.4d	−6.3	3.7
Ge	44.5a	44.0	52.7a	20.5	1.2	34.0b	14.5	54.5a	15.4	1.6	14.2c	−0.3	50.4a	16.8	3.6
Gf	25.0c	−19.2	39.0d	−10.9	1.6	38.3a	29.0	50.5b	7.0	1.3	16.9a	18.3	44.6b	3.3	2.6
Gg	29.5b	−4.5	40.2d	−8.1	1.4	25.2d	−15.2	49.0c	3.7	2.0	11.2d	−21.4	43.7bc	1.4	3.9
Gm	21.3d	−30.9	39.3d	−10.1	1.8	30.2c	1.7	51.5b	9.0	1.7	15.8ab	10.5	50.0a	16.0	3.2
Asp	43.3a	40.3	49.8b	13.9	1.2	24.5d	−17.5	47.5d	0.6	1.9	14.8bc	3.5	43.8bc	1.6	3.0
Control	30.9b		43.8c		1.4	29.7c		47.2d		1.6	14.3c		43.1c		3.0

**Notes.**

AMF species Gc
*Glomus clarum*
Ge
*G. etunicatum*
Gf
*G. fasciculatum*
Gg *Gigaspora* sp. Gm
*G.mosseae*
Asp *Acaulospora* sp

* Columns with a different letter on are significantly different, *n* = 3.

The polyphenol content was measured also in the tuber flesh of purple yams (Table 4). The polyphenol content in the methanol extracts from the tubers of purple flesh yams in the following treatment groups was statistically higher than in the control groups: Zihyuxieshu treated with Gm (30.0 mg/g) and Asp (23.3 mg/g), and Tainung 5 treated with Ge (18.9 mg/g), Gg (19.9 mg/g), and Gf (17.0 mg/g). Moreover, the polyphenol content in the tuber peel was higher than that in the tuber flesh.

**Table 4:** Two commercial species of purple flesh yams (*Dioscorea* spp.) were inoculated with six species of AMF. Polyphenol content of methanolic extracts from two species of purple flesh yams (*Dioscorea* spp.) flour inoculated with six species of AMF.

Polyphenol content of yams methanolic extract (mg/g)										
AMF species	Zihyuxieshu					Tainung 5				
	Tuber flesh	Increased rate (%)	Tuber peels	Increased rate (%)	Peels/Flesh ratio	Tuber flesh	Increased rate (%)	Tuber peels	Increased rate (%)	Peels/Flesh ratio
Gc	16.4e^*^	−22.9	44.3c	−1.7	2.7	15.2de	6.7	39.2b	10.1	2.6
Ge	19.2d	−9.6	45.1c	0.1	2.4	18.9b	32.6	41.7a	17.1	2.2
Gf	15.0f	−29.7	40.1d	−11.1	2.7	17.0c	19.2	34.5c	−3.1	2.0
Gg	21.0c	−1.5	47.9b	6.2	2.3	19.9a	39.6	38.8b	9.0	2.0
Gm	30.0a	41.2	51.6a	14.6	1.7	16.2cd	13.2	41.1a	15.4	2.5
Asp	23.3b	9.6	43.8c	−2.9	1.9	15.5d	8.2	39.5b	11.0	2.6
Control	21.3c		45.1c		2.1	14.3e		35.6c		2.5

**Notes.**

AMF species Gc
*Glomus clarum*
Ge
*G. etunicatum*
Gf
*G. fasciculatum*
Gg *Gigaspora* sp. Gm
*G. mosseae*
Asp *Acaulospora* sp

* Columns with a different letter on are significantly different, *n* = 3.

In the present study, increased concentration of total phenol in flesh and peels were found in the yams with higher AMF colonization levels than in the non-colonized control. In host-AMF interactions a range of defense mechanisms is activated in response to a microbial attack. Plant phenolics, particularly flavonoids and isoflavonoids are the most widespread classes of secondary metabolites known to be involved in the plant-microbe these interactions (Morandi, 1996). Glyceollin, shown to accumulated in soybean roots when mycorrhizal colonization was mature and well-developed (Morandi, Bailey & Gininazzipearson, 1984). An increased level of total phenols among plants with high mycorrhizal colonization levels could be due to the reaction of the plant against mycorrhizal colonization. Phenolic compounds accumulate in plant cells as a plant defense response against microorganisms (Vierheilig, 2004).

The root colonization by AMF enables host plants to produce high concentrations of phenolic compounds, thereby increasing their capacity for pest and disease resistance (Banuelos et al., 2014). The antioxidant capacity of plant extracts is derived from phenols, which are crucial for the antioxidant and biological activity of plants (Manthey, 2004; Aarti et al., 2013; Prieto & Vázquez, 2014). Yams are rich in polyphenols, which are secondary plant metabolites; the polyphenol content per 100 g of yams varies by species, ranging from 20 to 320 mg (Muzac-Tucker, Asemota & Ahamad, 1993). This study indicated that AMF inoculation positively affects the polyphenol content in yam tubers. All of the yam species tested had higher polyphenol content in the methanol extracts from the tuber peel than from the tuber flesh. Furthermore, as the polyphenol content in the tuber flesh increased, the content in the tuber peel also increased (Tables 3 and 4). Overall, AMF inoculation facilitates the synthesis and accumulation of polyphenols in yam tubers and enhances the antioxidant capacity of yams, rendering yams applicable for use in health foods.

### Flavonoid content

The flavonoid content in the methanol extracts from three species of white flesh yams inoculated with six species of AMF was evaluated (Table 5). The flavonoid content in the tuber flesh of the white yams in the following treatment groups was significantly higher than in the control group: Tainung 1 treated with Ge (7.5 mg/g) and Asp (8.6 mg/g); Tainung 2 treated with Ge (6.3 mg/g), Gf (6.9 mg/g), and Gm (6.4 mg/g); and Ercih treated with Gf (5.5 mg/g), Gm (5.4 mg/g), and Asp (5.6 mg/g). The results indicated that the flavonoid content in the tuber peel of the three white yams exceeded that found in the tuber flesh (Table 5).

**Table 5:** Three commercial species of white flesh yams (*Dioscorea* spp.) were inoculated with six species of AMF. Flavonoid content of methanolic extracts from three species of white flesh yams (*Dioscorea* spp.) flour inoculated with six species of AMF.

Flavonoid content of yams methanolic extract (mg/g)															
AMF species	Tainung 1					Tainung 2					Ercih				
	Tuber flesh	Increased rate (%)	Tuber peels	Increased rate (%)	Peels/Flesh ratio	Tuber flesh	Increased rate (%)	Tuber peels	Increased rate (%)	Peels/Flesh ratio	Tuber flesh	Increased rate (%)	Tuber peels	Increased rate (%)	Peels/Flesh ratio
Gc	6.1d^*^	−7.5	10.5d	3.8	1.7	4.4e	−9.2	10.2bc	3.2	2.3	4.5d	−7.9	7.8e	−17.7	1.7
Ge	7.5b	13.4	15.1a	49.1	2.0	6.3bc	29.3	11.3a	14.9	1.8	5.1b	3.8	10.0c	6.3	2.0
Gf	4.1f	−37.9	10.4d	2.0	2.5	6.9a	41.8	10.4b	5.5	1.5	5.5a	12.0	10.4b	10.8	1.9
Gg	6.0d	−9.2	9.9e	−2.9	1.6	6.1c	25.1	10.cd	0.9	1.6	4.8cd	−2.6	9.5d	1.3	2.0
Gm	4.8e	−27.3	11.9c	17.1	2.5	6.4b	31.8	10.4b	5.0	1.6	5.4a	10.6	10.9a	15.8	2.0
Asp	8.6a	28.7	12.7b	25.0	1.5	4.8d	−2.4	9.6e	−3.1	2.0	5.6a	14.0	10.4b	10.9	1.9
Control	6.6c		10.2de		1.5	4.9d		9.9d		2.0					1.9

**Notes.**

AMF species Gc
*Glomus clarum*
Ge
*G. etunicatum*
Gf
*G. fasciculatum*
Gg *Gigaspora* sp. Gm
*G. mosseae*
Asp *Acaulospora* sp

* Columns with a different letter on are significantly different, *n* = 3.

The flavonoid content in the tuber flesh of purple yams in the following treatment groups was significantly higher than in the control group: Zihyuxieshu treated with Ge (6.03 mg/g) and Gm (6.49 mg/g); and Tainung 5 treated with Gf (5.3 mg/g), Gg (5.7 mg/g), and Asp (5.2 mg/g) (Table 6). In several treatment groups such as Gc, Gg of Zihyuxieshu and Ge, Gm of Tainung 5 the flavonoid content in the tuber peel was two times higher than that in the tuber flesh (Table 6).

**Table 6:** Two commercial species of purple flesh yams (*Dioscorea* spp.) were inoculated with six species of AMF. Flavonoid content of methanolic extracts from two species of purple flesh yams (*Dioscorea* spp.) flour inoculated with six species of AMF.

Flavonoid content of yams methanolic extract (mg/g)										
AMF species	Zihyuxieshu					Tainung 5				
	Tuber flesh	Increased rate (%)	Tuber peels	Increased rate (%)	Peels/Flesh ratio	Tuber flesh	Increased rate (%)	Tuber peels	Increased rate (%)	Peels/Flesh ratio
Gc	5.0c^*^	0.5	11.5a	9.1	2.3	4.8c	10.4	8.3e	−13.0	1.7
Ge	6.0b	22.1	10.5b	−0.8	1.7	4.8c	8.8	10.0c	4.5	2.1
Gf	5.0c	0.7	9.3d	−12.1	1.9	5.3b	21.4	10.4b	9.6	2.0
Gg	5.0c	0.8	10.4b	−1.8	2.0	5.7a	29.8	9.6d	0.8	1.7
Gm	6.5a	31.4	11.8a	11.9	1.8	4.4d	0.8	10.9a	14.4	2.5
Asp	4.9c	−0.7	9.7c	−7.5	2.0	5.2b	18.6	10.1c	6.2	2.0
Control	4.9c		10.5b		2.1	4.4d		9.5d		2.2

**Notes.**

AMF species Gc
*Glomus clarum*
Ge
*G. etunicatum*
Gf
*G. fasciculatum*
Gg *Gigaspora* sp. Gm
*G. mosseae*
Asp *Acaulospora* sp

* Columns with a different letter on are significantly different, *n* = 3.

Most plants contain compounds such as flavonoids, which are secondary metabolites that serve as signaling molecules between plants and soil microorganisms (Antunes, Rajcan & Goss, 2006). Various studies have reported that flavonoids stimulate the colonization of AMF (Larose et al., 2002; Scervino et al., 2005), and that the amount of synthesized flavonoids increases before AMF colonize the plant roots (Larose et al., 2002). During the colonization process, flavonoids can facilitate spore germination and the extension of hyphae in plant roots. Additionally, the accumulation of isoflavones in plants is also associated with the growth of AMF (Scervino et al., 2005).

The concept of improving the contents respectively the yield of plant secondary metabolites through AMF is recent. For example, the accumulation of flavonoids compounds in medicinal plants colonized by AMF has been reported (Francineyde, Fábio & Leonor, 2014; Hemashenpagam & Selvaraj, 2011). Five medicinal herbs inoculated with three AMFs demonstrated that flavonoid contents were enhanced in all of the plants (Tejavathi & Jayashree, 2013). Flavonoids play a significant role in how plants interact with organisms, and have been proposed as regulatory compounds in the AM symbiosis (Morandi, 1996). Flavonoid levels in mycorrhizal plants are modulated by the developmental stage of the AM symbiosis (Harrison & Dixon, 1993). During root penetration and the establishment of the AM fungus in roots, intermediate levels of a number of flavonoids are detected, and at a late stage of root colonization, high levels of flavonoids, such as the phytoalexin medicarpin, can be found (Larose et al., 2002).

In this study, the inoculation of white yams with Ge, Gf, and Gm and that of purple yams with Gf, Gg, and Gm enhanced the generation of polyphenols and flavonoids in tubers. Similar to the polyphenol content results, all the tested yam species exhibited higher flavonoid content in the methanol extract from tuber peels than in the extract from tuber flesh. As the flavonoid content in the tuber flesh increased, the content in the tuber peel also increased. Thus, AMF inoculation facilitated the synthesis and accumulation of flavonoids in tuber peel. Because the antioxidant activity of flavonoids eliminates reactive oxygen and terminates the chain reaction of free radicals (Arora, Nair & Strasburg, 1998), AMF inoculation are predicted to enhance the health benefits of yams.

### Anthocyanin content

The anthocyanin content was measure in the tubers of purple yams inoculated with six species of AMF (Table 7). Compared with the control group, the inoculation of Zihyuxieshu with Gg (1.08 µmole/g) and Asp (0.97 µmole/g) promoted the accumulation of anthocyanins in tuber flesh, and inoculation with Gc (2.52 µmole/g), Ge (2.54 µmole/g), and Gm (2.47 µmole/g) boosted the anthocyanin content in tuber peel. Tainung 5 also exhibited elevated anthocyanin content in all treatment groups except for Gm. In particular, the anthocyanin content in the tuber flesh and peel of Tainung 5 inoculated with Gg (0.76 and 2.42 µmole/g) and Asp (0.69 and 2.38 µmole/g) was substantially and significantly higher than in the control group.

**Table 7:** Five commercial species of yams (*Dioscorea* spp.) were inoculated with six species of AMF. Anthocyanin content of methanolic extracts from five species of yams (*Dioscorea* spp.) tuber inoculated with six species of AMF.

	Anthocyanin content of yams methanolic extract (µmole/g)															
	Tainung 1		Tainung 2		Ercih		Zihyuxieshu					Tainung 5				
AMF species	Tuber flesh	Tuber peels	Tuber flesh	Tuber peels	Tuber flesh	Tuber peels	Tuber flesh	Increased rate (%)	Tuber peels	Increased rate (%)	Peels/Flesh ratio	Tuber flesh	Increased rate (%)	Tuber peels	Increased rate (%)	Peels/Flesh ratio
Gc	nd^**^	nd	nd	nd	nd	nd	0.86d^*^	3.6	2.52a	8.2	2.93	0.39e	5.4	1.83bc	1.7	4.69
Ge	nd	nd	nd	nd	nd	nd	0.76f	−8.4	2.54a	9.0	3.34	0.40d	8.1	1.88bc	4.4	4.70
Gf	nd	nd	nd	nd	nd	nd	0.70g	−15.7	2.25c	−3.4	3.21	0.45c	21.6	1.97b	9.4	4.38
Gg	nd	nd	nd	nd	nd	nd	1.08a	30.1	2.43ab	4.3	2.25	0.76a	105.4	2.42a	34.4	3.18
Gm	nd	nd	nd	nd	nd	nd	0.87c	4.8	2.47a	6.0	2.84	0.33g	−10.8	1.52d	−15.6	4.61
Asp	nd	nd	nd	nd	nd	nd	0.97b	16.9	1.93d	−17.2	1.99	0.69b	86.5	2.38a	32.2	3.45
Control	nd	nd	nd	nd	nd	nd	0.83e		2.33bc		2.81	0.37f		1.80c		4.87

**Notes.**

AMF species Gc
*Glomus clarum*
Ge
*G. etunicatum*
Gf
*G. fasciculatum*
Gg *Gigaspora* sp. Gm
*G.mosseae*
Asp *Acaulospora* sp

* Columns with a different letter on are significantly different, *n* = 3.

** nd, The anthocyanin composition could not be detected from the cultivars of Tainung 1, Tainung 2 and Ercih with white flesh.

Purple yams contain abundant anthocyanins, which are plant-specific polyphenolic compounds derived from flavonoid metabolism. The intermediate products generated during anthocyanin biosynthesis can help plants to resist the invasion of pathogens and environmental adversities (Padmavati et al., 1997). Furthermore, anthocyanins also have antioxidant properties (Wei et al., 2011) and are able to reduce LDLs, prevent cardiovascular diseases, inhibit lipid peroxidation, resist the attack of free radicals, and diminish oxidative stress (Frankel et al., 1998).

This study revealed that AMF inoculation may influence the metabolic pathway of anthocyanin in purple yams, specifically those inoculated with Gg and Asp, thereby increasing the anthocyanin content in the two purple yam species. Furthermore, the anthocyanin content in the tuber peel of the purple yam species was several times higher than that in the tuber flesh, where an increase in flavonoid content was followed by an increase in anthocyanin content (Tables 6 and 7). We postulate that because the cortical tissues of the host plant are the area on which AMF symbiotically associates, and the mycorrhizal structure may activate the biosynthesis of anthocyanins in yam peels, thereby influencing the accumulation of anthocyanins.

### Evaluation of yam food quality

The comparison between tuber weight increase rate and flavonoids, anthocyanin content demonstrated changes of two species of purple flesh yams by inoculated with six AMF species (Table 8). For Zihyuxieshu yams, excluding the Asp inoculation treatment, the tuber weight of all AMF-inoculated Zihyuxieshu yams were higher than those of the controls. The Zihyuxieshu yams inoculated with Ge and Gm exhibited markedly increased flavonoids production of flesh by 22.1% and 31.4% and inoculated with Gf, Asp and Gg increased anthocyanin production of flesh by 15.7%, 16.9% and 30.1% respectively. For Tainung 5 yams, the tuber weight of all AMF-inoculated Tainung 5 yams were higher than those of the controls. The Tainung 5 yams inoculated with Asp, Gf and Gg exhibited markedly increased flavonoids production of flesh by 18.6%, 21.4% and 29.8% and increased anthocyanin production of flesh by 86.5%, 21.6% and 105.4% respectively. These species are recommended for further research regarding their effectiveness to improve yam productivity and quality in the study area.

**Table 8:** AMF inoculation on tuber weights and secondary metabolite content in yam tubers. The comparability between of tuber weight and flavonoid, anthocyanin increase rate of the two species of purple flesh yams by inoculated with six AMF species.

AMF species	Zihyuxieshu					Tainung 5				
	Tuber increased rate (%)	Flesh flavonoids increased rate (%)	Peels flavonoids increased rate (%)	Flesh anthocyanin increased rate (%)	Peels anthocyanin increased rate (%)	Tuber increased rate (%)	Flesh flavonoids increased rate (%)	Peels flavonoids increased rate (%)	Flesh anthocyanin increased rate (%)	Peels anthocyanin increased rate (%)
Gc	59.4	0.5	9.1	3.6	8.2	59.7	10.4	−13.0	5.4	1.7
Ge	56.5	22.1	−0.8	8.4	9.0	40.1	8.8	4.5	8.1	4.4
Gf	33.3	0.7	−12.1	15.7	3.4	13.9	21.4	9.6	21.6	9.4
Gg	73.5	0.8	−1.8	30.1	4.3	36.4	29.8	0.8	105.4	34.4
Gm	5.9	31.4	11.9	4.8	6.0	28.3	0.8	14.4	10.8	15.6
Asp	−1.4	−0.7	−7.5	16.9	17.2	7.1	18.6	6.2	86.5	32.2

**Notes.**

AMF species Gc
*Glomus clarum*
Ge
*G. etunicatum*
Gf
*G. fasciculatum*
Gg *Gigaspora* sp. Gm
*G. mosseae*
Asp *Acaulospora* sp

In summary, symbiotic AMF can induce changes in the accumulation of secondary metabolites (Yao et al., 2003). During the establishment of AMF symbiosis, a range of chemical and biological parameters is affected in plants, including the pattern of secondary plant compounds. The accumulation of flavonoids and phenolic compounds in plants colonized by AMF has been reported (Devi & Reddy, 2002; Larose et al., 2002). We tested a wide variety of species of yams and AMF to look for significant interactions in chemical compounds. This study represents an effect of AMF on secondary metabolites important to healthy crops. Our five hosts responded differently to AMF, demonstrating both positive and negative response to AMF inoculation on the phenolic and anthocyanin level. From an agri-food industry perspective, it may be valuable to explore the extent of multifunctionality among AMF, and whether this may play a role in enhancing crop quality and food value, or may be potentially enhance human health.

## Conclusion

The results of this study showed that all six AMF species were able to form a symbiotic relationship with the five species of host yams and enhance the yam tuber yield. Yam tubers can produce considerable amounts of medicinal secondary metabolites. The inoculation of yams with AMF influenced the generation and content of secondary antioxidant metabolites, namely, polyphenols, flavonoids, and anthocyanins. The positive effect of AMF on plants can boost the yield of yams and the quality of the secondary metabolites, thus enhancing the antioxidant capacity of yams, making them applicable for use in health food. Moreover, yam peel is typically regarded as processing waste. However, this study proved that ample functional ingredients, involving polyphenols, flavonoids, and anthocyanins, can be extracted from the peel, thereby increasing the potency and utilization of yam tubers. In this study, the data show Tainung 1, Tainung 2 and Ercih three white flesh yams species treated with Ge and Gm enhanced the yam tuber yield and influenced the polyphenols and flavonoids content. Two species of purple yams, Zihyuxieshu and Tainung 5, treated with Ge and Gg enhance the yam tuber yield and influenced the polyphenols, flavonoids and anthocyanins content. These species are recommended for further research about their effectiveness to improve yam productivity and quality. The symbiotic affinity between AMF species and host yams manifested to varying degrees; thus, in practical cultivation, the AMF species that has a superior symbiotic relationship with yams should be selected and applied in yam inoculation to achieve optimal field cultivation. The inoculation of yams with AMF can expedite plant growth and increase the content of secondary metabolites in tubers. Therefore, AMF can be used as a microbial fertilizer and developed for yam cultivation and production.

## Supplemental Information

10.7717/peerj.1266/supp-1 Table S1 Statistics description Three replicates were examined for each treatment based on 10 plant root sections (1 cm) for each replicate. The mycorrhizal formation rates of the various AMF species were calculated based on the percentage of the root segments colonized by AMF.

10.7717/peerj.1266/supp-2 Table S2 Statistics description

10.7717/peerj.1266/supp-3 Table S3 Statistics description The tuber flesh and peel polyphenol content in the methanol extracts from three species of white flesh yams inoculated with six species of AMF was measured. The three species yams were each inoculated with one of the six different AMF species. The control group was not subjected to inoculation. Thus, this experiment comprised seven treatments, each with three replicates. Data were analyzed by one way ANOVA using SAS 9.1 Statistic program. Difference between treatments were determined using Dancan’s Multiple Range Test (*P* < 0.05).

10.7717/peerj.1266/supp-4 Table S4 Statistics description The tuber flesh and peel polyphenol content in the methanol extracts from two species of purple flesh yams inoculated with six species of AMF was measured. The two species yams were each inoculated with one of the six different AMF species. The control group was not subjected to inoculation. Thus, this experiment comprised seven treatments, each with three replicates. Data were analyzed by one way ANOVA using SAS 9.1 Statistic program. Difference between treatments were determined using Dancan’s Multiple Range Test (*P* < 0.05).

10.7717/peerj.1266/supp-5 Table S5 Statistics description The tuber flesh and peel flavonoid content in the methanol extracts from three species of white flesh yams inoculated with six species of AMF was measured. The three species yams were each inoculated with one of the six different AMF species. The control group was not subjected to inoculation. Thus, this experiment comprised seven treatments, each with three replicates. Data were analyzed by one way ANOVA using SAS 9.1 Statistic program. Difference between treatments were determined using Dancan’s Multiple Range Test (*P* < 0.05).

10.7717/peerj.1266/supp-6 Table S6 Statistics description The tuber flesh and peel flavonoid content in the methanol extracts from two species of purple flesh yams inoculated with six species of AMF was measured. The two species yams were each inoculated with one of the six different AMF species. The control group was not subjected to inoculation. Thus, this experiment comprised seven treatments, each with three replicates. Data were analyzed by one way ANOVA using SAS 9.1 Statistic program. Difference between treatments were determined using Dancan’s Multiple Range Test (*P* < 0.05).

10.7717/peerj.1266/supp-7 Table S7 Statistics description The tuber flesh and peel Anthocyanin content from five species of yams inoculated with six species of AMF was measured. The five species yams were each inoculated with one of the six different AMF species. The control group was not subjected to inoculation. Thus, this experiment comprised seven treatments, each with three replicates. But the anthocyanin composition could not be detected from the cultivars of Tainung 1, Tainung 2 and Ercih with white flesh. Data were analyzed by one way ANOVA using SAS 9.1 Statistic program. Difference between treatments were determined using Dancan’s Multiple Range Test (*P* < 0.05).
