# Supplementary material for: The influence of arbuscular mycorrhizal fungi inoculation on yam (Dioscorea spp.) tuber weights and secondary metabolite content
Source: PeerJ. 2015 Sep 24;3:e1266. doi: 10.7717/peerj.1266 (PMC4586806; doi:10.7717/peerj.1266)
Supplement: Table S1 — Three replicates were examined for each treatment based on 10 plant root sections (1 cm) for each replicate. The mycorrhizal formation rates of the various AMF species were calculated based on the percentage of the root segments colonized by AMF. [file peerj-03-1266-s001.doc]

Table 2 Statistics description.

Three replicates were examined for each treatment based on 10 plant root sections (1cm) for each replicate. The mycorrhizal formation rates of the various AMF species were calculated based on the percentage of the root segments colonized by AMF.

| mycorrhizal formation rate (%) | | | | | |
| --- | --- | --- | --- | --- | --- |
| AMF species | Tainung 1 | Tainung 2 | Ercih | Zihyuxieshu | Tainung 5 |
| *Glomus clarum*（Gc） | 80％  80％  40％ | 80％  90％  50％ | 100％  90％  70％ | 50％  60％  90％ | 70％  60％  90％ |
| *G. etunicatum*（Ge） | 100％  90％  70％ | 90％  90％  70％ | 80％  80％  100％ | 80％  80％  90％ | 100％  80％  60％ |
| *G. fasciculatum*（Gf） | 70％  90％  60％ | 100％  100％  70％ | 80％  60％  100％ | 100％  70％  90％ | 90％  50％  90％ |
| *Gigaspora*sp.（Gg） | 100％  50％  80％ | 80％  90％  80％ | 70％  60％  90％ | 100％  70％  70％ | 100％  70％  90％ |
| *G. mosseae*（Gm） | 100％  70％  100％ | 100％  100％  60％ | 50％  90％  60％ | 100％  70％  90％ | 60％  50％  80％ |
| *Acaulospora* sp（Asp） | 90％  100％  70％ | 50％  70％  90％ | 100％  90％  70％ | 90％  90％  80％ | 80％  80％  60％ |
| control | 0％  10％  10％ | 10％  10％  0％ | 10％  20％  10％ | 10％  20％  0％ | 20％  10％  10％ |
