# Supplementary material for: The influence of arbuscular mycorrhizal fungi inoculation on yam (Dioscorea spp.) tuber weights and secondary metabolite content
Source: PeerJ. 2015 Sep 24;3:e1266. doi: 10.7717/peerj.1266 (PMC4586806; doi:10.7717/peerj.1266)
Supplement: Table S2 [file peerj-03-1266-s002.doc]

Table 2 Statistics description.

The comparison of tuber weights and size between the AMF-inoculated and noninoculated yams after 8 months of cultivation. The five species yams seedlings were each inoculated with one of the six different AMF species. The control group was not subjected to inoculation. Thus, this experiment comprised seven treatments, each with five replicates. Data were analyzed by one way ANOVA using SAS 9.1 Statistic program. Difference between treatments were determined using Dancan’s Multiple Range Test (P＜0.05).

| Tuber weight (g/plant) | | | | | |
| --- | --- | --- | --- | --- | --- |
| AMF species | Tainung 1 | Tainung 2 | Ercih | Zihyuxieshu | Tainung 5 |
| *Glomus clarum*（Gc） | 1790  2000  2120  1700  2050 | 2200  2170  1960  2110  2170 | 2110  1940  1410  1530  1890 | 1520  2200  2140  1850  1490 | 880  860  1880  1060  1420 |
| *G.etunicatum*（Ge） | 1960  1990  2140  1820  2270 | 2310  2040  2070  2810  1830 | 2000  1860  1630  1580  2370 | 2290  1650  1830  1490  1770 | 1230  830  840  1190  1260 |
| *G.fasciculatum*（Gf） | 1525  1690  1790  1950  1610 | 2170  2440  2480  2410  2910 | 1970  1680  1530  1970  1320 | 1520  1430  1270  1590  1880 | 1070  740  670  980  890 |
| *Gigaspora*sp.（Gg） | 1600  1380  1730  1550  1430 | 1940  2020  2090  2310  2440 | 1150  1690  1640  1550  1140 | 2380  2190  1910  1640  1890 | 930  890  1240  980  1170 |
| *G.mosseae*（Gm） | 2020  1610  1650  1580  1910 | 2620  2410  2180  2160  2490 | 1540  1600  1860  2340  1860 | 1460  1010  1270  980  1390 | 770  1180  920  1040  990 |
| *Acaulospora* sp（Asp） | 1470  1240  1720  1430  1580 | 1580  1630  1690  1300  1840 | 1400  1750  1380  1790  1770 | 1390  970  860  1190  1280 | 890  780  670  820  930 |
| control | 1300  1190  1320  1710  1780 | 1640  1530  1420  1710  1890 | 1770  1680  1620  1250  1520 | 1410  1100  1240  990  1030 | 620  880  790  720  810 |
