# Supplementary material for: The influence of arbuscular mycorrhizal fungi inoculation on yam (Dioscorea spp.) tuber weights and secondary metabolite content
Source: PeerJ. 2015 Sep 24;3:e1266. doi: 10.7717/peerj.1266 (PMC4586806; doi:10.7717/peerj.1266)
Supplement: Table S3 — The tuber flesh and peel polyphenol content in the methanol extracts from three species of white flesh yams inoculated with six species of AMF was measured. The three species yams were each inoculated with one of the six different AMF species. The control group was not subjected to inoculation. Thus, this experiment comprised seven treatments, each with three replicates. Data were analyzed by one way ANOVA using SAS 9.1 Statistic program. Difference between treatments were determined using Dancan’s Multiple Range Test (P < 0.05). [file peerj-03-1266-s003.doc]

Table 3. Statistics description

The tuber flesh and peel polyphenol content in the methanol extracts from three species of white flesh yams inoculated with six species of AMF was measured. The three species yams were each inoculated with one of the six different AMF species. The control group was not subjected to inoculation. Thus, this experiment comprised seven treatments, each with three replicates. Data were analyzed by one way ANOVA using SAS 9.1 Statistic program. Difference between treatments were determined using Dancan’s Multiple Range Test (P＜0.05).

| AMF species | Polyphenol content of yams methanolic extract（mg/g） | | | | | |
| --- | --- | --- | --- | --- | --- | --- |
|  | Tainung 1 | | Tainung 2 | | Ercih | |
|  | Tuber  flesh | Tuber  peels | Tuber  flesh | Tuber  peels | Tuber  flesh | Tuber  peels |
| *Glomus clarum*（Gc） | 31.64  27.98  31.17 | 50.01  50.25  48.98 | 18.56  20.20  20.71 | 43.73  44.90  43.17 | 10.26  11.29  11.71 | 40.22  41.34  39.70 |
| *G.etunicatum*（Ge） | 44.90  44.72  43.83 | 51.75  53.81  52.59 | 35.34  32.86  33.84 | 54.75  55.08  53.58 | 13.45  14.29  14.90 | 50.72  50.39  50.06 |
| *G.fasciculatum*（Gf） | 23.71  24.84  26.34 | 39.75  38.43  38.72 | 39.84  37.17  38.01 | 51.28  50.39  49.92 | 17.34  17.06  16.21 | 44.62  45.00  44.06 |
| *Gigaspora* sp.（Gg） | 30.00  29.81  28.68 | 39.47  40.97  40.12 | 24.42  25.78  25.40 | 48.23  49.03  49.59 | 10.59  11.34  11.71 | 43.45  44.43  43.31 |
| *G.mosseae*（Gm） | 19.92  21.84  22.26 | 39.70  39.47  38.81 | 29.06  30.37  31.22 | 51.70  51.00  51.65 | 15.93  15.51  15.84 | 50.67  50.25  49.12 |
| *Acaulospora* sp.（Asp） | 42.51  44.15  43.31 | 50.39  49.59  49.45 | 22.96  25.03  25.59 | 46.31  47.29  48.84 | 13.87  14.29  16.12 | 44.29  43.50  43.68 |
| control | 30.89  31.50  30.28 | 41.81  45.14  44.29 | 31.07  29.11  28.97 | 46.50  47.90  47.25 | 13.82  14.29  14.67 | 42.42  43.12  43.83 |
