# Supplementary material for: The influence of arbuscular mycorrhizal fungi inoculation on yam (Dioscorea spp.) tuber weights and secondary metabolite content
Source: PeerJ. 2015 Sep 24;3:e1266. doi: 10.7717/peerj.1266 (PMC4586806; doi:10.7717/peerj.1266)
Supplement: Table S7 — The tuber flesh and peel Anthocyanin content from five species of yams inoculated with six species of AMF was measured. The five species yams were each inoculated with one of the six different AMF species. The control group was not subjected to inoculation. Thus, this experiment comprised seven treatments, each with three replicates. But the anthocyanin composition could not be detected from the cultivars of Tainung 1, Tainung 2 and Ercih with white flesh. Data were analyzed by one way ANOVA using SAS 9.1 Statistic program. Difference between treatments were determined using Dancan’s Multiple Range Test (P < 0.05). [file peerj-03-1266-s007.doc]

Table 7. Statistics description

The tuber flesh and peel Anthocyanin content from five species of yams inoculated with six species of AMF was measured. The five species yams were each inoculated with one of the six different AMF species. The control group was not subjected to inoculation. Thus, this experiment comprised seven treatments, each with three replicates. But the anthocyanin composition could not be detected from the cultivars of Tainung 1, Tainung 2 and Ercih with white flesh. Data were analyzed by one way ANOVA using SAS 9.1 Statistic program. Difference between treatments were determined using Dancan’s Multiple Range Test (P＜0.05).

|  | Anthocyanin content of yams methanolic extract（mg/g） | | | | | | | | | |
| --- | --- | --- | --- | --- | --- | --- | --- | --- | --- | --- |
|  | Tainung 1 | | Tainung 2 | | Ercih | | Zihyuxieshu | | Tainung 5 | |
| AMF species | Tuber  flesh | tuber  peels | Tuber  flesh | Tuber  peels | tuber  flesh | Tuber  peels | Tuber  flesh | tuber  peels | Tuber  flesh | Tuber  peels |
| *Glomus clarum*（Gc） | nd | nd | nd | nd | nd | nd | 0.857  0.859  0.861 | 2.475  2.622  2.464 | 0.389  0.393  0.395 | 1.848  1.894  1.759 |
| *G.etunicatum*（Ge） | nd | nd | nd | nd | nd | nd | 0.754  0.757  0.758 | 2.503  2.531  2.584 | 0.398  0.400  0.400 | 1.860  1.895  1.871 |
| *G.fasciculatum*（Gf） | nd | nd | nd | nd | nd | nd | 0.693  0.696  0.696 | 2.291  2.258  2.212 | 0.445  0.444  0.447 | 2.002  1.943  1.959 |
| *Gigaspora* sp.（Gg） | nd | nd | nd | nd | nd | nd | 1.081  1.086  1.088 | 2.383  2.416  2.477 | 0.759  0.769  0.765 | 2.366  2.440  2.448 |
| *G.mosseae*（Gm） | nd | nd | nd | nd | nd | nd | 0.866  0.874  0.872 | 2.444  2.472  2.499 | 0.332  0.323  0.3224 | 1.516  1.503  1.527 |
| *Acaulospora* sp.（Asp） | nd | nd | nd | nd | nd | nd | 0.964  0.968  0.970 | 2.054  1.874  1.866 | 0.692  0.689  0.697 | 2.343  2.439  2.346 |
| control | nd | nd | nd | nd | nd | nd | 0.826  0.833  0.831 | 2.367  2.335  2.281 | 0.378  0.370  0.372 | 2.010  1.620  1.772 |
